# Is the Association between Herbal Use and Blood-Pressure Control Mediated by Medication Adherence? A Cross-Sectional Study in Primary Care

**DOI:** 10.3390/ijerph182412916

**Published:** 2021-12-07

**Authors:** Phaviga Thangsuk, Kanokporn Pinyopornpanish, Wichuda Jiraporncharoen, Nida Buawangpong, Chaisiri Angkurawaranon

**Affiliations:** 1 Department of Family Medicine, Faculty of Medicine, Chiang Mai University, Chiang Mai 50200, Thailand; phaviga@gmail.com (P.T.); wichudaj131@gmail.com (W.J.); nidalooknum@gmail.com (N.B.); chaisiri.a@cmu.ac.th (C.A.); 2 Global Health and Chronic Conditions Research Group, Chiang Mai University, Chiang Mai 50200, Thailand

**Keywords:** behavioral medicine, patient adherence, hypertension, primary care, complementary and alternative medicine

## Abstract

Herbs have been used worldwide for many health conditions as an alternative treatment, including hypertension. Their use might affect the use of conventional medications, as well as blood-pressure control. This study aims to determine whether the potential associations between herb use and high blood pressure in hypertensive patients was mediated by medication adherence. A cross-sectional study was conducted using questionnaires and available medical databases at a primary care clinic of a tertiary hospital in Chiang Mai, Thailand. The data were collected from 450 patients with essential hypertension. Drug adherence was assessed by the Morisky Green Levine Medication Adherence Scale. The history of herbs used in the past three months was obtained. The goal of controlled blood pressure was defined in accordance with the Thai guidelines on the treatment of hypertension. Of the total 450 patients, 42% had high adherence. Nearly 18% reported herb use in the past three months. High medication adherence was strongly associated with blood-pressure control when adjusted for age, gender, education, the presence of comorbidities, and herb use (aOR 26.73; 95% CI 8.58–83.23; *p* < 0.001). The association between herb use and blood-pressure control did not achieve statistical significance (*p* = 0.143). However, the adjusted odds ratio of the association between herb use and blood-pressure control was diluted from 0.67 to 0.83 when adding the factor of medication adherence to the model. In conclusion, herb use was associated with poor medication adherence, which was in turn associated with poor blood-pressure control. Assessing this information contributes to appropriate exploration and counseling.

## 1. Introduction

Hypertension is one of the serious global health problems. High blood pressure is a major factor of coronary artery disease, cerebrovascular disease, and other comorbidities, such as heart failure, chronic kidney disease, and blindness in diabetic patients, which cause disability and premature death [1]. Taking antihypertensive medication regularly is crucial to control blood pressure to effectively decrease and prevent any potential comorbidities [2].

According to the World Health Organization (WHO), in developed countries, 30–50% of patients with non-communicable diseases take medication regularly, and the problem increases in circumstances of lower attention to healthcare and public health services [3,4]. Taking medicine is considered a healthy habit that is correlated with personal beliefs and knowledge about diseases and treatments [5]. Many studies have been reported on medication adherence and the associated factors [6,7,8]. Some believe that alternative treatment may interfere with adherence to conventional medication. Herbal use is one of the concerns [9,10,11,12].

Presently, herbs are used worldwide as an alternative treatment for many health conditions. One is to control cardiovascular disease, in approximately 18% of the population [13]. Herbs can come in a variety of foods, beverages, and multivitamin supplements [14]. The motives for use of herbs include the understanding of their benefit to health, acceptance that they are from nature, not the chemical products that can be toxic to the body, and the history of the herbal therapy [15]. Herbs have also been perceived by Thai people to be beneficial to health as medicine [16]. 

People tend to use herbal medication believing that it can help make them healthier. Some people believe that it can be used together with the conventional medication that they receive from the hospital [17]. However, there is an unclear indication that herb use can treat cardiovascular disease [18]. Herb use might reduce or substitute for the use of conventional medications [17]. There is an increasing risk of potential herb–drug interactions, adverse reactions, and contraindications. Moreover, their use is difficult to monitor, and the consequences of use are inadequately known [14,19]. With limited evidence on the data of correlation between herb use and medication adherence among hypertensive patients, this study aims to examine whether the potential associations between herb use and high blood pressure was mediated by medication adherence. The three specific research questions were: 

(1) Is herb use and medication adherence associated with hypertension control?

(2) Is there an association between herb use and medication adherence?

(3) Is the association between herb use and hypertension control mediated by medication adherence?

## 2. Materials and Methods

### 2.1. Design and Setting

This research is a cross-sectional study collecting data from adults with essential hypertension treated at a primary care clinic in Maharaj Nakorn Chiang Mai Hospital (Chiang Mai University Hospital), which is a tertiary hospital located in the city center of Chiang Mai, Thailand. 

### 2.2. Patients

Our screening nurses invited patients aged 35 years old and over, who had been diagnosed with essential hypertension, and were taking at least one antihypertensive drug, to participate in the study. Walk-in patients were recruited using a convenience sampling method. Patients who did not communicate in the Thai language, or had secondary hypertension, were excluded from the study. All participants fitting the inclusion criteria were invited until the target sample size was reached. There were 558 patients invited to participate and 450 were included for final analysis (19.4% decline to participate). All patients provided written informed consent to the study. 

### 2.3. Data Collection

We used the two-independent-proportion formula to calculate the sample size. The calculation was conducted based on the results from prior study [11]. The proportion of patients with good blood-pressure control among good adherence compared to those with poor adherence were 0.636 and 0.497, respectively. To reach a power of 80% and alpha value of 0.05, the estimated size sample was 426. We then decided to take 450 samples. 

The data were collected by self-report questionnaires and past medical history reviewed from electronic medical records. The questionnaire comprised general information, medical history about hypertensive care and hospitalization related to hypertension, and medication through the past three months including antihypertensive drugs, the experience of side effects of the drug, and medication adherence assessment. A trained research nurse helped explain some questions which might be problematic. Herbal use was assessed by self-report questions about their experience within the past three months. The definition of herbal medicine was following the WHO statement which includes herbs, herbal materials, herbal preparations, and finished herbal products that contain, as active ingredients, parts of plants, or other plant materials, or combinations [20]. Patients who reported the regular use of oral form herbal medication for any purpose were categorized as “use”.

The secondary data from the electronic medical records of the hospital database were body mass index value, prescribed medicine in the last three months (generic name, number of tablets, times per day) and medical history of hypertensive complication treatment or emergency care related to hypertension.

### 2.4. Measures

#### 2.4.1. Medication Adherence Measures

Morisky Green Levine Medication Adherence Scale, a four-item questionnaire, was used for evaluating medication adherence with a “yes” or “no” response for each question. The results were categorized into three groups including high adherence (4 scores), moderate adherence (2–3 scores), and low adherence (0–1 score) [21]. The author (C.A.) received the original MGL questionnaire from the Morisky Medication Adherence Research, LLC to use and translate. The translation of the questionnaire followed the WHO suggested standard procedure. The validation study has not been performed for the Thai language but has been used in published research [22]. Factor analysis was performed and the factor loadings of all MGL items ranged from 0.54 to 0.74 (>0.4). The study sample showed a Cronbach’s Alpha of 0.53 which was consistent with the derivation study (0.61) [23].

#### 2.4.2. Blood-Pressure Control

To assess the achievement in blood-pressure control, participants had automatic blood-pressure device measurements with the same device throughout the study. Each person had blood pressure measured twice with a 15-min interval. Then the average value was collected for analysis. The blood pressure was defined in accordance with the Thai guidelines on the treatment of hypertension by the Thai Hypertension Society [24]. The goals were (1) below 140/90 mmHg for under 80-year-old patients, (2) below 150/90 mmHg for patients aged 80 years or older, or (3) below 130/80 mmHg for patients with HF with albuminuria over 30 milligrams per day. 

### 2.5. Statistical Analysis 

The data were analyzed with STATA version 15.1 (Stata Corp LCC, College Station, TX, USA). Chi-square and ANOVA were used to analyze factors of medication adherence. Association between herb use, medication adherence, and blood-pressure control was analyzed using logistic regression analysis. To demonstrate the mediator effect, according to the Baron and Kenny method for mediation analysis [25], it should fit the following criteria: (1) significant association between independent variable and mediator, (2) significant association between independent variable and the dependent variable, (3) significant association between mediator and dependent variable, and (4) reduction of impact of the independent variable on the dependent variable after controlling for the mediator. Variables including age, sex, and education were priori confounders and always included in the multivariable model. For other potential confounders, we used a backward step wise approach to reduce the number of variables. Calibration of the model was assessed with the Hosmer–Lemeshow goodness-of-fit test. A *p*-value of less than 0.05 was considered to have statistical significance.

## 3. Results

Of the total 450 patients, more than half were female (59.11%). The average age was 64.96 years old (SD 9.04). Almost all participants were of Thai nationality (99.11%). The average time of the first hypertensive diagnosis was 9.93 years (SD 5.77). About 90% of the patients had comorbidities. Nearly 18% used herbal medicine in the past three months.

Table 1 shows that patients with low medication adherence were 26 (5.78%), moderate adherence were 235 (52.22%) and high adherence were 189 (42%). From the univariable analysis, the data suggested that mean blood pressure differed between different categories of medication adherence (*p* < 0.001). The mean systolic blood pressure among those with low adherence was 147.27 mmHg (SD 19.71) while the mean systolic blood pressure among those with high adherence was 134.63 mmHg (SD 12.60). This was similar to the finding for mean diastolic blood pressure, 80.75 mmHg (SD 12.81) for low adherence and 72.57 mmHg (SD 10.61) for high adherence. The correlation table is included in Supplementary Table S1.

According to Table 2, the association between herb use and blood-pressure control did not achieve statistical significance (OR 0.67, *p* = 0.143). However, the adjusted odds ratio was diluted to 0.83 (*p* = 0.534) when adding the factor of medication adherence to the model.

## 4. Discussion

Around 42% of the study population reported high medication adherence in our study. This study suggested that low adherence was statistically significant when associated with blood-pressure control and herbal medicine impacting medication adherence in the pathway of blood-pressure control.

Not surprisingly, compared to research from other countries, we found the similar proportion of high medication adherence in our population. Although most studies used different tools to assess medication adherence, studies in Asian countries also reported a similar trend [11,26,27,28,29]. For example, a study in a public primary care clinic in Singapore using the Medication Adherence Report Scale-5 (MARS-5) reported that about 55% of the participants had high medication adherence [28]. A study in Malaysia using the seven-item validated survey form for medication adherence reported that around 53% had good adherence [29].

In the association between herb use and medication adherence, the study indicated that patients who take herbal medicines tend to have lower medication adherence. Meanwhile, the result does not correlate with a cross-sectional study of 300 patients in a geriatrics clinic in the US that did not reveal the correlation between herb use and medication adherence [30]. This is in accordance with a previous study in Brazil [31]. Patients with hypertension who took medical herbs tended to have lower medication adherence and this is interactive to the adherence and dosage of the herb used. It is assumed that patients chose herbal medicine to substitute for the conventional medication, which was the result of medication myths [17]. Believing that conventional medicine causes negative effects on the body, side effects or complications from medical maltreatment as well as inconvenience, side effects, poor doctor-patient relationship and high cost of medicine impelled patients to choose alternative medicine and poor medication adherence [31,32].

Low medication adherence correlates to the failure of blood-pressure control, which is also illustrated in this study [33]. When the influence of herb use was studied in the realm of medication adherence, herbal medicine seems not to influence the treatment directly, yet has an impact on the adherence. When medication adherence was adjusted in the analysis, the relationship between the disease control and herb use decreased. This might imply that patients who take herbal medicine but take conventional medicine irregularly would have poor disease control. Therefore, an evaluation of herbal medication should go hand in hand with an evaluation of conventional medication adherence as well as knowledge and worry about the medicine as the resource for further relevant actions.

The strength of this study is that we both interviewed and reviewed the health information from each patient’s medical record. Therefore, high reliability of data should be obtained. However, there are some limitations to this study. First, histories about healthcare, such as the number of medications, history of admission, or history of emergency room visits, were assessed based on the secondary data in the database of Chiang Mai University Hospital. Therefore, the healthcare services that patients received elsewhere were not included. However, the self-report on obtaining these kinds of services in other places was not reported in any case. Secondly, to assess the herb use, we did not limit the purpose of use only for hypertension control, as generally Thai people used it for their general health believing that it would help for overall disease control. Thirdly, the level of medication adherence was assessed solely based on the self-report MGL Adherence Scale, and was not compared with the assessment carried out objectively. However, our study has provided reliability and factor validity of the questionnaire in our results. Fourthly, the samples were patients in a single primary care unit. Samples in other contexts such as rural area may reveal different results. Lastly, previous literature examining herb use and hypertension control that is similar to our setting was difficult to find. The sample size of this study was calculated based on more reliable estimates between adherence and hypertension control. This might be a explanation of why our study may be underpowered when detecting some associations. The direction on associations seen does suggest that there may be a mediation effect, and that larger studies are needed.

## 5. Conclusions

Although the criteria for mediation analyses was not met, we demonstrated that low medication adherence influences blood-pressure control and that herb use was associated with poor adherence. Consequently, improving medication adherence may be necessary in those patients using herbs. Individual assessments regarding background knowledge and beliefs on mainstream medicine and alternative medicine is needed for further intervention. Previous studies indicate that despite worrying about the medicine, the patients still cooperate with the treatment if they are properly informed [34,35]. Additionally, patients and physicians should cooperate to ease any occurring problems to enhance medication adherence that results from other factors.

## Figures and Tables

**Table 1:** Patient characteristics stratified by medication adherence.

	Low Adherence N = 26	Moderate Adherence N = 235	High Adherence N = 189	*p*-Value
**Patient Characteristics**				
Female gender, n(col%)	12 (46.15)	134 (57.02)	120 (63.49)	0.155
Age (years), mean ± SD	62.00 ± 7.89	64.14 ± 9.38	66.40 ± 8.55	0.008
Thai nationality, n(col%)	26 (100)	235 (100)	185 (97.88)	0.062
Education level, n(col%)				
Secondary school and lower	15 (57.69)	130 (55.32)	126 (66.67)	
Higher than secondary school	11 (42.31)	105 (44.68)	63 (33.33)	0.058
Health Insurance, n(col%)				
No	2 (7.69)	18 (7.66)	18 (9.52)	
Yes	24 (92.31)	217 (92.34)	171 (90.48)	0.782
Presence of Co-morbidity				
None, n(col%)	4 (15.38)	23 (9.79)	18 (9.52)	0.639
Diabetes mellitus, n(col%)	8 (30.77)	64 (27.23)	38 (20.11)	0.176
Dyslipidemia, n(col%)	21 (80.77)	203 (86.38)	157 (83.07)	0.546
IHD, n(col%)	0 (0)	3 (1.28)	3 (1.59)	0.799
CVD, n(col%)	1 (3.85)	11 (4.68)	3 (1.59)	0.209
Hyperthyroid, n(col%)	1 (3.85)	1 (0.43)	2 (1.06)	0.200
Chronic kidney disease, n(col%)	5 (19.23)	40 (17.02)	21 (11.11)	0.184
Caregiver involved in medication management, n(col%)	3 (11.54)	10 (4.26)	14 (7.45)	0.185
Family History of hypertension, n(col%)	12 (46.15)	150 (63.83)	114 (60.64)	0.204
Family History of CVD or IHD, n(col%)	6 (23.08)	52 (22.13)	26 (13.76)	0.075
History of admission due to hypertensive emergency condition, n(col%)	5 (19.23)	23 (9.79)	12 (6.35)	0.075
History of ED visit due to HTN-related condition, n(col%)	2 (7.69)	17 (7.23)	10 (5.29)	0.695
Years taken drug, mean ± SD	8.11 ± 4.79	9.85 ± 5.63	10.30 ± 6.05	0.184
Daily dose frequency, mean ± SD	1.81 ± 0.49	1.82 ± 0.48	1.85 ± 0.51	0.806
Number of drugs taken, mean ± SD	3.79 ± 1.96	3.46 ± 2.10	3.70 ± 2.16	0.467
Experience of side effects, n(col%)	4 (15.38)	29 (12.34)	15 (7.94)	0.249
Herbal use, n(col%)	11 (42.31)	40 (17.09)	29 (15.34)	0.003
**Treatment Outcome**				
Controlled BP, n(col%)	5 (19.23)	123 (52.34)	160 (84.66)	<0.001
Systolic BP, mean ± SD	147.27 ± 19.71	139.86 ± 15.50	134.63 ± 12.60	<0.001
Diastolic BP, mean ± SD	80.75 ± 12.81	76.29 ± 10.35	72.57 ± 10.61	<0.001

**Table 2:** The association between herb use, medication adherence, and blood-pressure control using logistic regression analysis.

	Univariable Analysis		Model 1 *		Model 2 **	
Variables	Crude OR	*p*-Value	Adjusted OR	*p*-Value	Adjusted OR	*p*-Value
Medication adherence						
Low	1.00				1.00	
Moderate	4.61	0.003	-	-	5.10	0.003
High	23.17	<0.001			26.73	<0.001
Age	1.01	0.592	1.02	0.139	1.01	0.623
Gender						
Female	1.00		1.00		1.00	
Male	1.03	0.879	0.84	0.451	0.75	0.247
Presence of co-morbidity						
Diabetes mellitus	0.19	<0.001	0.17	<0.001	0.16	<0.001
Cerebrovascular disease	0.19	0.006	0.14	0.002	0.16	0.007
Chronic kidney disease	0.30	<0.001	0.28	<0.001	0.30	<0.001
Education						
More than secondary	1.00		1.00		1.00	
Less than secondary	0.90	0.608	0.81	0.413	0.93	0.780
Herbal use	0.72	0.188	0.67	0.143	0.83	0.534

* Model 1: adjusted for age, gender, co-morbidity, education (Hosmer–Lemeshow test: χ^2^ = 5.16, df = 8, *p*-value = 0.739); ** Model 2: adjusted for age, gender, co-morbidity, education, medication adherence (Hosmer–Lemeshow test: χ^2^ = 11.80, df = 8, *p*-value = 0.160).

## Data Availability

The data underlying this article will be shared on reasonable request to the corresponding author.

## Supplementary Materials

The following are available online at https://www.mdpi.com/article/10.3390/ijerph182412916/s1, Table S1: Correlations between each variable.
