# Supplementary material for: Is the Association between Herbal Use and Blood-Pressure Control Mediated by Medication Adherence? A Cross-Sectional Study in Primary Care
Source: Int J Environ Res Public Health. 2021 Dec 7;18(24):12916. doi: 10.3390/ijerph182412916 (PMC8702107; doi:10.3390/ijerph182412916)
Supplement: Supplementary file 1 [file ijerph-18-12916-s001.zip › ijerph-1475088-supplementary.pdf]

Supplementary Table S1. Correlations between each variable.

	MGL score	Herbs use	SBP	DBP
MGL score	1.000			
Herbs use	-0.106*	1.000		
SBP	-0.217**	-0.002	1.000	
DBP	-0.235**	0.049	0.470**	1.000

*p-value <0.05, **p-value <0.001

Abbreviations: MGL, Morisky Green Levine; SBP, systolic blood pressure; DBP, diastolic blood pressure
